# Fluorodeoxyglucose uptake in laryngeal carcinoma is associated with the expression of glucose transporter-1 and hypoxia-inducible-factor-1α and the phosphoinositide 3-kinase/protein kinase B pathway

**DOI:** 10.3892/ol.2014.1877

**Published:** 2014-02-12

**Authors:** KUI ZHAO, SHU-YE YANG, SHUI-HONG ZHOU, MENG JIE DONG, YANG-YANG BAO, HONG-TIAN YAO

**Affiliations:** 1 Department of PET Center, The First Affiliated Hospital, College of Medicine, Zhejiang University, Hangzhou, Zhejiang 310003, P.R. China; 2 Department of Otolaryngology, The First Affiliated Hospital, College of Medicine, Zhejiang University, Hangzhou, Zhejiang 310003, P.R. China; 3 Department of Pathology, The First Affiliated Hospital, College of Medicine, Zhejiang University, Hangzhou, Zhejiang 310003, P.R. China

**Keywords:** laryngeal carcinoma, glucose transporter-1, hypoxia inducible factor-1α, phosphoinositide 3-kinase/protein kinase B signal pathway, fluorodeoxyglucose, prognosis

## Abstract

High fluorodeoxyglucose (FDG) uptake by human carcinomas, including head and neck cancers, is associated with a poor prognosis. Glucose transporter-1 (Glut-1) is believed to be an intrinsic marker of hypoxia in malignant tumors. The expression of hypoxia-inducible factor-1α (HIF-1α) and correlated target genes, including Glut-1, is regulated by the phosphoinositide 3-kinase/protein kinase B (PI3K/Akt) pathway. However, it remains unclear whether the PI3K/Akt signaling pathway is involved in regulating FDG uptake directly. In the present study, 24 consecutive patients with laryngeal carcinoma were examined pre-operatively and the standardized uptake values (SUV) of the laryngeal carcinomas were determined. Glut-1, HIF-1α, PI3K and phosphorylated-Akt (p-Akt) expression was detected by immunohistochemical staining of paraffin sections from the tumor specimens. Associations among SUV_max_, Glut-1, HIF-1α, PI3K and p-Akt protein expression and the other clinical parameters were analyzed. The univariate analyses revealed a significantly shorter survival time along with higher HIF-1α (P=0.018) and PI3K (P=0.008) expression, but the survival time was not significantly correlated with Glut-1 or p-Akt expression. The multivariate analysis demonstrated that higher SUV_max_ (P=0.043) and PI3K expression (P=0.012) were significantly correlated with a poor survival time. Spearman’s rank analysis showed significant correlations between SUV_max_ and HIF-1α (r=0.577; P=0.003), PI3K (r=1.0; P<0.0001) and p-Akt (r=0.577; P=0.003) expression. PI3K was associated with poorly- and moderately-differentiated laryngeal carcinoma (P=0.012). In conclusion, a high SUV_max_ indicates a poor prognosis for laryngeal carcinoma. Also, a high SUV_max_ may be associated with the increased expression of Glut-1, HIF-1α, PI3K and p-Akt.

## Introduction

[^18^F]-2-fluoro-2-deoxy-D-glucose (^18^F-FDG) positron emission tomography/computed tomography (PET/CT) imaging has been used widely for the diagnosis, pre-operative staging, restaging, prognostic prediction and detection of unknown primary tumors (1–3). Increased uptake of FDG, a glucose analog, directly reflects the higher glucose metabolic rate in malignant tumor cells compared with their non-malignant counterparts (4,5).

Numerous mechanisms have been proposed for the accelerated glucose use in growing tumors and in transformed and malignant cells, including passive diffusion, Na^+^-dependent glucose transport, oncogenes and facilitative glucose transporter (Glut) (6–8). Numerous studies (9–12), including our previous study (6), have revealed that Glut-1 plays a significant role in malignant glucose metabolism and that it may contribute to increased FDG uptake. Certain studies have considered Glut-1 as a possible intrinsic marker of hypoxia in malignant tumors (13,14). Hypoxia of solid tumors has been associated with therapy resistance and a poor clinical prognosis. Biological markers that predict tumor hypoxia may be useful for selecting treatments and predicting patient prognosis (9). Certain studies have demonstrated that FDG indirectly reflects the hypoxic status of malignant tumors (9,10,15–18) since FDG is associated with hypoxia markers, including Glut-1, phosphoinositide 3-kinase (PI3K) and hypoxia inducible factor-1α (HIF-1α) (2,9,10,15–17). High FDG uptake by human carcinomas is also associated with a poor prognosis (9,19). Our previous study identified that the FDG uptake detected by single photon emission CT in head and neck cancers was associated with increased Glut-1 expression (20). Our recent preliminary study also revealed that the maximum standardized uptake value (SUV_max_) of PET/CT in the cervical lymph nodes predicted cervical metastasis of a carcinoma from an unknown primary tumor (3). There is certain controversy, however, as certain studies did not find any association between FDG uptake and the hypoxic status of specific carcinomas (21,22). The expression of HIF-1α and the correlated target genes, including Glut-1, is regulated by the PI3K/protein kinase B (Akt) pathway (23,24). However, it remains unclear whether the PI3K/Akt signal pathway is involved in the regulation of FDG uptake (15,16,25,26). Consequently, the association between FDG and hypoxia markers must be studied further. Therefore, the correlation between FDG uptake and the hypoxia markers, Glut-1, HIF-1α, PI3K and Akt, was investigated in the present study using immunohistochemistry to clarify whether FDG-PET can be used to predict tumor hypoxia and the prognosis of patients with laryngeal carcinoma.

## Materials and methods

### Patients

Using PET/CT, 24 consecutive patients [21 male and 3 female; mean age, 60.8 years (range, 37–80 years)] with laryngeal carcinoma were examined pre-operatively. No patient received tumor-directed therapy pre-operatively. The institutional review board of The First Affiliated Hospital, College of Medicine, Zhejiang University (Hangzhou, Zhejiang, China) approved the present study, and written informed consent was obtained from each patient prior to inclusion.

### PET/CT

Whole-body imaging was performed using a combined PET/CT scanner (Biograph Sensation 16; Siemens Medical, Erlangen, Germany). Following ≥4–6 h of fasting, the patients were administered an intravenous injection of FDG at a dosage of 5.5–7.4 MBq (0.15–0.20 mCi)/kg. The blood glucose levels of the patients were checked prior to the ^18^F-FDG injection. Data acquisition started 60–90 min after FDG administration following routine procedures: Low-dose CT was performed from head to mid-thigh at 120 kV, 50 mA and with a 2–5-mm slice thickness matching the PET section, followed by three-dimensional PET scanning at 2 min/bed position. The PET emission scan was performed from the subcranial region to mid-thigh, whereas the brain scan required another bed position. The PET images were reconstructed iteratively using ordered subset Syngo Speaking (Wizard Workstation; Siemens Medical Solutions USA, Inc., Malvern, PA, USA) with CT-based attenuation correction. PET, CT and fused PET/CT images were generated and reviewed on a workstation. The SUV_max_ was assessed from the predominant lesion and calculated based on the amount of injected ^18^F-FDG and the body weight: SUV = [decay corrected activity (kBq)/tissue volume (ml)]/[injected FDG activity (kBq)/body weight (g)] (3). The PET/CT images were interpreted independently by two experienced PET/CT physicians blinded to the clinical data. Any variations between the interpretation of the data were settled by consensus.

### Immunohistochemical analysis and evaluation

For the immunohistochemical evaluation, paraffin blocks of formalin-fixed specimens were obtained via biopsies of the predominant lesions in each subject. Formalin-fixed, paraffin-embedded tissue blocks from the primary lesions were cut into 4-μm sections, and representative sections were analyzed immunohistochemically (EliVision™ Plus IHC kit; Fuzhou Maixin Biotechnology Development, Co., Ltd., Fuzhou, Fuijan, China), using a rabbit polyclonal antibody against Glut-1 (1:50), a mouse monoclonal antibody against HIF-1α (1:100), a rabbit monoclonal antibody against PI3K (1:100) and a rabbit polyclonal antibody against p-AKT (1:50) (all Santa Cruz Biotechnology, Inc., Santa Cruz, CA, USA) (25). Briefly, antigen retrieval was performed subsequent to the sections being deparaffinized with xylene and dehydrated through an ethanol series. Endogenous peroxidase activity was blocked by incubating the slides in 1.5% hydrogen peroxide in absolute methanol at room temperature for 10 min. Primary antibodies were applied for 1 h at room temperature, followed by use of 50 μl polymer enhancer for 20 min and 50 μl polymerized horseradish peroxidase-anti-mouse immunoglobulin G for 30 min. The reaction products were visualized using a diaminobenzidine kit (Fuzhou Maixin Biological Technology, Ltd.), and then the sections were counterstained with hematoxylin and eosin, dehydrated and examined under a light microscope. Tris-buffered saline was used instead of the primary antibody for the negative controls. Erythrocytes, which were present in all sections, served as internal controls for Glut-1 to confirm the constant immunostaining intensity.

Glut-1, HIF-1α, PI3K and p-Akt were evaluated by the same investigator who was blinded to the clinical and follow-up data. Glut-1 expression was considered positive only if distinct membrane staining was present. HIF-1α, PI3K and p-AKT proteins were observed in the nucleus and cytoplasm. Protein analysis was performed in 10 random high fields, and 100 tumor cells were counted in each high-power field for each case and for all antibodies used. The percentage of positive cells was calculated by dividing the number of positive tumor cells by the total number of tumor cells counted. A sample was considered negative if <25% of the cells were stained (2).

### Follow-up

The patients were asked to come back for follow-up at 1-, 3-, and 6-month intervals in the first, second and third to fifth years after the initial surgery, respectively, and then annually thereafter. Follow-up consisted of a routine physical examination, strobolarynoscopy and CT or magnetic resonance imaging of the primary site. Patient follow-up was reported up to the date of the last visit to the clinic.

### Statistical analyses

The statistical analyses were performed using SPSS for Windows, version 19.0 (SPSS, Inc., Chicago, IL, USA). Associations among SUV_max_, Glut-1, HIF-1α, PI3K and p-Akt protein expression and the other pre-treatment parameters were analyzed using the χ^2^ test and Fisher’s exact test. Logistic regression was used for the multivariate analysis. P<0.05 was deemed to indicate statistically significant differences, and the correlation analysis was performed using Spearman’s correlation.

## Results

### Patients’ characteristics

The clinicopathological findings, including age, gender, site, tumor-node-metastasis (TNM) stage, pathological type, recurrence, metastasis and follow-up, are listed in Table I. Of the 24 patients, 22 (91.7%) presented with squamous cell carcinomas and two (8.3%) with neuroendocrine carcinomas. In total, 19 (79.2%), four (16.7%), and one (4.2%) patient exhibited tumors located in the glottis, supraglottis and subglottis, respectively. All patients underwent surgery and eight (33.3%) received post-operative radiotherapy. The average follow-up period was 33.0 months (range, 6–63 months). During the follow-up, two patients (8.3%) were lost, three (12.5%) developed local recurrence and two (8.3%) developed distant metastases. At the last follow-up, 14 patients (58.3%) remained alive. The median overall survival time (OS) was 42.3 months [95% confidence interval (CI), 32.3–52.2 months). The three-year survival rate was 59.3%.

### SUV_max_ and survival analysis

**Figure 1:**
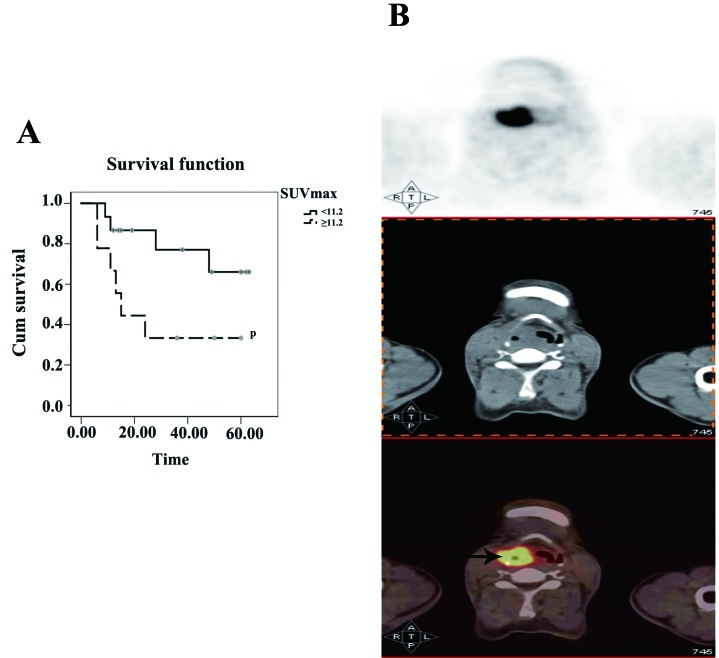
(A) Significant differences in mean survival time between the higher (≥11.2) and lower (<11.2) SUV_max_ subgroups were determined by univariate analysis (P=0.05). The multivariate analysis showed that SUV_max_ was significantly associated with a poor prognosis (P=0.043). (B) The high SUV_max_ in PET/CT. SUV, standardized uptake value; PET/CT, positron emission tomography/computed tomography.

No significant difference in SUV_max_ was found between pathological types, TNM stages, differentiation status and tumor sites in the patients. The mean SUV_max_ was 11.2 (range, 6.2–23.9). When the mean SUV_max_ was defined as the cutoff, there was a significant difference in mean survival time between the higher (≥11.2, n=9) and lower (<11.2, n=15) SUV_max_ subgroups (28.3 vs. 50.9 months; P=0.05) (Fig. 1).

### Expression of Glut-1, HIF-1α, PI3K and p-Akt

**Figure 2:**
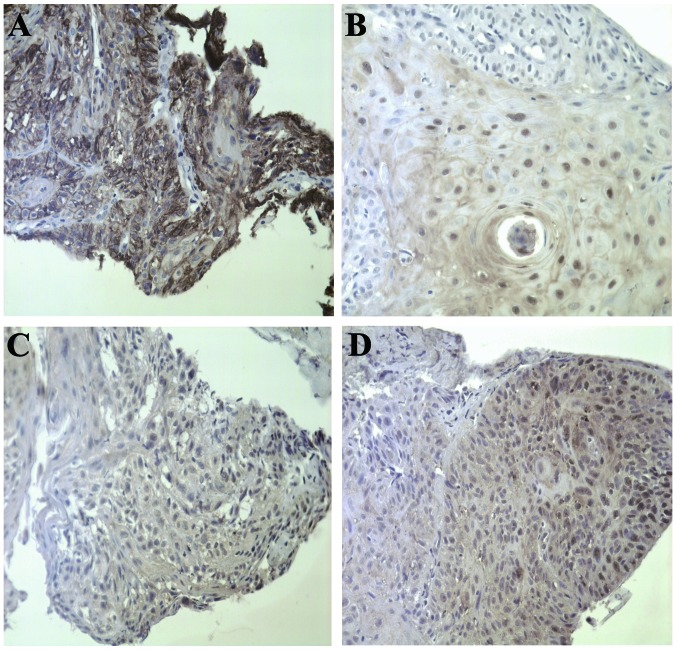
Immunohistochemical staining showing that (A) Glut-1 staining occurred diffusely in the membranes of the cancer cells, (B) HIF-1α occurred in a granular and diffuse pattern localized mainly in the cytoplasm of the cancer cells and (C) PI3K and (D) p-AKT proteins were detected in the nucleus and cytoplasm (EliVision, ×40). HIF-α hypoxia-inducible factor-1α; PI3K, phosphoinositide 3-kinase; Glut-1, glucose transporter-1; p-AKT, phosho-protein kinase B.

In the present study, 58.3% (14/24), 50.0% (12/24), 29.2% (7/24) and 54.2% (13/24) of the laryngeal carcinomas were positive for Glut-1, HIF-1α, PI3K and p-Akt protein, respectively (Table I; Fig. 2).

Spearman’s rank analysis showed significant correlations between the expression of Glut-1 and HIF-1α (r=0.676; P<0.001), Glut-1 and PI3K (r=0.418; P=0.042), Glut-1 and p-Akt (r=0.580; P=0.003), HIF-1α and PI3K (r=0.707; P<0.001), HIF-1α and p-Akt (r=0.753; P<0.001) and PI3K and p-Akt (r=0.650; P=0.001).

### Correlation between SUV_max_ and hypoxic markers

Spearman’s rank analysis showed significant correlations between SUV_max_ and Glut-1 (r=0.577; P=0.003), HIF-1α (r=1.0; P<0.0001), PI3K (r=1.0; P<0.0001) and p-Akt (r=0.577; P=0.003) expression.

**Figure 3:**
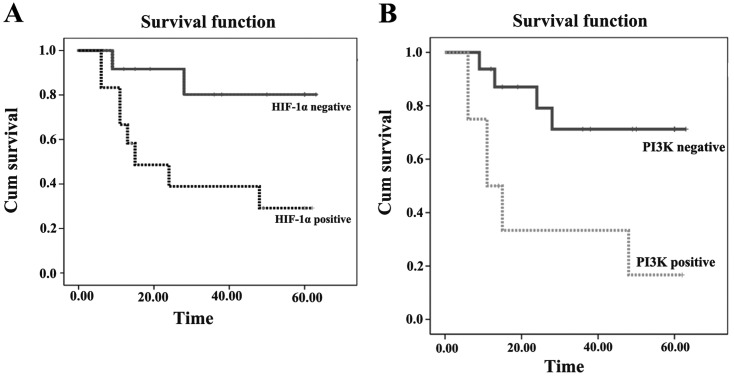
(A) HIF-1α and (B) PI3K expression were significantly associated with a poorer survival in the univariate analyses (P=0.018 and P=0.008, respectively). HIF-α, hypoxia-inducible factor-1α; PI3K, phosphoinositide 3-kinase.

The associations between Glut-1, HIF-1α, PI3K and p-Akt expression and the clinicopathological features are shown in Table I. The univariate analyses revealed a significantly shorter survival time in those patients with high HIF-1α expression compared with negative HIF-1α expression (30.4 vs. 54.5 months; P=0.018; Fig. 3A). There was also a significant correlation between survival time and the expression of PI3K (P=0.008; Fig. 3B). By contrast, survival time was not correlated with Glut-1 or p-Akt expression. The multivariate analysis showed that SUV_max_ (P=0.043) and PI3K (P=0.012) were significantly associated with a poor prognosis.

The expression of Glut-1, HIF-1α and p-Akt was not significantly associated with any clinicopathological factor (gender, age, pathological type, differentiation, TNM, site, recurrence and metastasis). However, significantly higher PI3K expression was observed in poorly- and moderately-differentiated laryngeal carcinomas compared with well-differentiated carcinomas (P=0.012). PI3K expression was not significantly associated with the other clinicopathological factors of gender, age, pathological type, TNM, site, recurrence and metastasis.

## Discussion

Previous studies have demonstrated that FDG uptake may be an independent prognostic factor in head and neck tumors in general, including for certain patients with laryngeal cancer (3,19,27). In our previous study, it was found that the SUV_max_ of PET/CT in cervical lymph nodes may predict cervical metastasis of carcinoma from an unknown primary tumor (25 patients) (3). In the present study, it was revealed that the mean survival time for those with a low SUV_max_ (<11.2) was much longer than those with a high SUV_max_ (≥11.2) (50.9 vs. 28.3 months, P=0.05). However, the association between SUV and the prognosis of head and neck cancer continues to be debated (28,29). Haerle *et al* did not find a significant correlation between SUV_max_ and a higher prevalence of metastasis, or as a surrogate for a worse outcome (28). Schinagl *et al* identified that the integrated SUV was associated with local control and survival time, while SUV_mean_ and SUV_max_ were not (29). These variations are believed to be due to use of varying treatments, a range of SUV cutoff values and the heterogeneity of tumor sites (27–29).

FDG uptake is also associated with specific molecular markers. Of these, Glut-1 plays an important role in the increased FDG uptake in cancers (9–11,15). However, no correlation between FDG uptake and Glut-1 expression has been observed in patients with colorectal (16) or head and neck cancer (29). Therefore, the FDG uptake in cancer tissues may involve a complicated glucose-metabolizing pathway, and Glut-1 may not be the key factor in the pathway (16). Certain studies have revealed that other molecular markers, including the upstream regulators of Glut-1 and HIF-1α (2,9), or the PI3K/Akt pathway (15,26,30), are involved in the process of FDG uptake. Bos *et al* found that there are positive correlations between FDG uptake and Glut-1 expression, mitotic activity index, amount of necrosis, number of tumor cells/volume, expression of hexokinase I, number of lymphocytes and microvessel density (18). To the best of our knowledge, the present study is the first to evaluate the correlations between SUV and Glut-1, HIF-1 and the PI3K/Akt pathway in laryngeal carcinoma. There were significant correlations between SUV_max_ and Glut-1 (r=0.577; P=0.003), HIF-1α (r=1.0; P<0.0001), PI3K (r=1.0; P<0.0001) and p-Akt (r=0.577; P=0.003) expression, indicating that a high FDG uptake was significantly associated with a poor outcome in laryngeal carcinoma. The expression of HIF-1α and PI3K was associated with survival time in the univariate analyses, and PI3K continued to be a prognostic factor in the multivariate analysis, while the other markers (Glut-1 and p-Akt) were not directly associated with survival time in patients with laryngeal carcinoma. Conversely, no significant difference was found in SUV_max_ according to pathological type, TNM stage, differentiation or tumor site. In addition, the expression of Glut-1, HIF-1α, PI3K and p-Akt was not associated with these clinicopathological factors, other than PI3K being associated with poorly- and moderately-differentiated laryngeal carcinoma. Therefore, a correlation between the molecular basis of FDG uptake and these hypoxic markers was indicated; this concurs with the study by Kaira *et al,* which reported significant associations between FDG activity and the expression of Glut1, HIF-1α, hexokinase I, vascular endothelial growth factor, cluster of differentiation 34, Ki-67, mammalian target of rapamycin (mTOR) and p53 in malignant pleural mesothelioma. The PI3K/AKT/mTOR signaling pathway may play a crucial role in the glycolytic system associated with FDG uptake (15).

In the present study, the significant interdependence among Glut-1 and HIF-1α, and PI3K and p-Akt, indicated that Glut-1 is regulated by HIF-1 and the PI3K/Akt pathway. In our recent study, it was demonstrated that Glut-1 expression was correlated with the expression of PI3K and p-Akt in 42 patients with head and neck adenoid cystic carcinoma (31). The PI3K/Akt pathway promotes Glut-1 cell-surface trafficking and activity (32). Not only are the activation and phosphorylation of PI3K/Akt well-recognized regulators of cell growth, survival outcomes and angiogenesis, they also play significant roles in promoting glucose metabolism (33). AKT activation may be responsible for metabolic processes during the Warburg effect (15). Melstrom *et al* identified that PI3K inhibitors downregulated Glut-1 mRNA and protein expression, and were involved in mediating Glut-1 activity (32).

HIF-1α may be involved in PI3K/Akt regulation. In addition to its role as a glucose transporter, Glut-1 is a factor in the cellular response to hypoxia as a downstream target of HIF-1α. The HIF complex then binds to hypoxia-responsive elements in target genes and activates their transcription. The PI3K/Akt pathway has been indicated in the control of HIF-1α protein expression and Glut-1 expression (23,33). Burrows *et al* demonstrated that the PI3K inhibitor, GDC-0941, reduced the HIF-1α, p-AKT and Glut-1 expression in thyroid carcinoma cells *in vitro* and *in vivo* (34).

The present study has several limitations, including the small number of patients and the variations in treatments and primary sites. Therefore, further investigation of the correlation between FDG uptake and hypoxic markers *in vivo* and *in vitro* is required.

Due to the small sample size, a definitive conclusion cannot be drawn, however, the results indicate that a high SUV_max_ predicts a poor prognosis in laryngeal carcinoma. A high SUV_max_ may also be associated with increased Glut-1, HIF-1α, PI3K and p-Akt expression. The study indicates that PET/CT can be used as a marker of tumor hypoxia and the prognosis of patients with laryngeal carcinoma. Further study is required to confirm these findings.

## Figures and Tables

**Table I:** Clinicopathological characteristics of 24 patients with laryngeal carcinoma.

Pt no.	Gender/age, years	Pathology	HG	Site	TNM	Treatment	Follow-up, months	Glut-1	HIF	PI3K	p-Akt	SUV_max_
1	M/58	NC	PD	Subglottic	T2N2M0	TL+SND +PRT	6 R, 18 DOD	+	+	+	+	17.3
2	M/67	NC	PD	Supraglottic	T2N2cM0	TL+SND +PRT	8 R, 10 M, 15 DOD	+	+	+	+	15.5
3	F/64	SCC	WD	Glottic	T1N0M0	LC 60 NED	_	_	_	_	6.8	
4	M/69	SCC	WD	Glottic	T1bN0M0	FPL	60 NED	+	_	_	+	8.7
5	M/63	SCC	WD	Glottic	T2N0M0	VPL	60 NED	-	-	-	+	7.0
6	M/75	SCC	WD	Glottic	T1N0M0	LC	62 NED	+	+	-	+	8.5
7	M/57	SCC	WD	Glottic	T1N0M0	LC	63 NED	-	-	-	-	8.4
8	M/64	SCC	WD	Glottic	T3N0M0	TL	60 NED	+	+	-	+	15.0
9	M/80	SCC	WD	Supraglottic	T2N2M0	TL	12 R, 24 DOD	+	+	-	-	22.0
10	M/49	SCC	MD	Glottic	T1N0M0	VPL	24 NED	+	_	_	_	7.8
11	M/65	SCC	MD	Glottic	T3N0M0	TL+PRT	48 lost	-	+	+	+	10.3
12	M/37	SCC	PD	Supraglottic	T2N1M0	NPL	14 NED	+	+	+	+	8.3
13	M/55	SCC	WD	Glottic	T1N0M0	LC	15 NED	-	-	-	-	6.2
14	M/59	SCC	MD	Glottic	T4N0M0	TL+PRT	11 DOD	+	+	+	+	10.5
15	M/71	SCC	MD	Glottic	T2N0M0	TL+PRT	13 DOD	+	+	-	+	12.5
16	M/38	SCC	WD	Glottic	T1N0M0	LC	19 NED	-	-	-	-	7.3
17	M/64	SCC	MD	Glottic	T3N0M0	TL+PRT	6 DOD	+	+	+	+	18.7
18	M/61	SCC	PD	Glottic	T3N0M0	TL+PRT	49 NED	+	+	-	+	7.9
19	M/67	SCC	MD	Glottic	T4N2M0	TL+PRT	11 DOD	+	+	+	+	23.9
20	M/51	SCC	WD	Glottic	T3N1M0	TL+PRT	50 NED	-	-	-	-	11.2
21	M/65	SCC	MD	Glottic	T2N0M0	TL+PRT	28 DOD	-	-	-	-	9.1
22	F/69	SCC	WD	Glottic	T1N0M0	LC	9 lost	-	-	-	-	7.7
23	M/61	SCC	WD	Supraglottic	T1N0M0	HPL	24 lung M, 36 AWD	+	-	-	-	11.6
24	F/49	SCC	WD	Glottic	T1N0M0	LC	38 NED	-	-	-	-	6.9

Pt no., patient number; TNM, tumor-node-metastasis; Glut-1, glucose transporter-1; HIF, hypoxia-inducible factor; PI3K, phosphoinositide 3-kinase; p-Akt; phosphorylated-protein kinase B; SUV, standardized uptake values; NC, neuroendocrine carcinoma; SCC, squamous cell carcinoma; HG, histological grade; WD, well-differentiated; MD, moderately-differentiated; PD, poorly-differentiated; TL, total laryngectomy; FPL, frontal partial laryngectomy; VPL, vertical partial laryngectomy; HPL, horizontal partial laryngectomy; NTL, near total laryngectomy; SND, selective neck dissection; PRT, post-operative radiotherapy; LC, laryngofissure cordectomy; R, recurrence; M, metastasis; DOD, died of disease; NED, no evidence of disease; AWD, alive with disease.
